# New Thrombotic Events in Ischemic Stroke Patients with Elevated Factor VIII

**DOI:** 10.1155/2014/302861

**Published:** 2014-12-17

**Authors:** Brittany M. Gouse, Amelia K. Boehme, Dominique J. Monlezun, James E. Siegler, Alex J. George, Katherine Brag, Karen C. Albright, T. Mark Beasley, Cindy Leissinger, Ramy El Khoury, Sheryl Martin-Schild

**Affiliations:** ^1^Stroke Program, Department of Neurology, Tulane University School of Medicine, 1440 Canal Street, TB-52, Suite 1000, New Orleans, LA 70112-2715, USA; ^2^Gertrude H. Sergievsky Center, Department of Neurology, Columbia University, 630 West 168 Street, New York, NY 10032, USA; ^3^Stroke Program, Department of Neurology, Hospital of the University of Pennsylvania, 3400 Spruce Street, 2nd Floor, Ravdin Building, Philadelphia, PA 19104, USA; ^4^Department of Epidemiology, School of Public Health, University of Alabama at Birmingham, 1665 University Boulevard, Birmingham, AL 35294, USA; ^5^Health Services and Outcomes Research Center for Outcome and Effectiveness Research and Education (COERE), 1530 3rd Avenue South, Medical Towers, Birmingham, AL 35294, USA; ^6^Center of Excellence in Comparative Effectiveness Research for Eliminating Disparities (CERED), Medical Towers Building, 1717 11th Avenue South, Suite 516A, Birmingham, AL 35294, USA; ^7^Section on Statistical Genetics, Department of Biostatistics, School of Public Health, University of Alabama at Birmingham, 1665 University Boulevard, Birmingham, AL 35294, USA; ^8^Section of Hematology/Oncology, Department of Medicine, Tulane University School of Medicine, 1430 Tulane Avenue, New Orleans, LA, USA

## Abstract

*Background*. Heightened levels of Factor VIII (FVIII) have been associated with both arterial and venous thrombosis. While elevated FVIII is common during acute ischemic stroke (AIS), whether elevated FVIII confers an increased risk for recurrent thrombotic events (RTEs) following AIS has not been previously explored. *Methods*. Consecutive AIS patients who presented to our center between July 2008 and September 2013 and had FVIII measured during admission were identified from our stroke registry. Baseline characteristics and the occurrence of RTE (recurrent or progressive ischemic stroke, DVT/PE, and MI) were compared in patients with and without elevated FVIII levels. *Results*. Of the 298 patients included, 203 (68.1%) had elevated FVIII levels. Patients with elevated FVIII had higher rates of any in-hospital RTE (18.7% versus 8.4%, *P* = 0.0218). This association remained after adjustment for baseline stroke severity and etiology (OR 1.01, 95% CI 1.00–1.01, *P* = 0.0013). Rates of major disability were also higher in patients who experienced a RTE (17.8% versus 3.2%, *P* < 0.0001). *Conclusion*. A significantly higher frequency of in-hospital RTEs occurred in AIS patients with elevated FVIII. The occurrence of such events was associated with higher morbidity. Further study is indicated to evaluate whether FVIII is a candidate biomarker for increased risk of RTEs following AIS.

## 1. Introduction

The procoagulant Factor VIII (FVIII) plays an important role in the activation of thrombin and ultimately in the formation of a fibrin-rich thrombus. Elevated plasma levels of FVIII have been previously associated with an increased risk of both venous [1–3] and arterial thrombotic events [4–7]. Elevated plasma levels of FVIII have been found to be common in patients with acute ischemic stroke (AIS) [6, 7], one of the leading causes of death and significant long-term disability worldwide [8].

Previous prospective work has found plasma FVIII levels to be one of the strongest hemostatic predictors of ischemic stroke occurrence [6]. More recent research has demonstrated elevated levels of plasma FVIII to be present in approximately 70% of patients with AIS during hospital admission, suggesting further that elevated FVIII levels are common during the acute phase of ischemic stroke [7]. FVIII has also been shown to be associated with other thromboembolic conditions known to complicate the course of AIS, such as deep vein thrombosis (DVT) [2], pulmonary embolism (PE) [3], and myocardial infarction [4, 5] in several different patient populations. Despite evidence suggesting an association between elevated FVIII levels and the occurrence of thromboembolism, whether there exists any association between elevated FVIII levels and the occurrence of new thrombotic events following AIS has not been previously explored.

The purpose of this study was to investigate the relationship between elevated FVIII levels and recurrent thrombotic events during hospitalization for AIS. We hypothesized that new thrombotic events would occur more frequently in patients with elevated FVIII levels than in patients with normal FVIII levels.

## 2. Methods

### 2.1. Study Population

Patients who presented to our medical center with an AIS between July 2008 and September 2013 were evaluated using a comprehensive Institutional Review Board (IRB) approved, prospective registry [9]. Patients were included if they were admitted to our stroke service with a clinical diagnosis of AIS. Patients were diagnosed with AIS through clinical history and exam, supported by neuroimaging methods. Patients who did not have their FVIII evaluated during their stroke admission or who were below the age of 18 at the time of admission were excluded.

Stroke etiology was determined using the Trial of Org 10172 in Acute Stroke Treatment (TOAST) classification [10]. In all patients, the TOAST classification National Institutes of Health Stroke Scale (NIHSS) score and modified Rankin Scale (mRS) score were determined by certified practitioners. Additional data not collected as a routine part of the registry was collected with an IRB approved chart review by trained abstractors blinded to FVIII level at the time of collection. Included patients were divided into two groups according to our center's laboratory reference range for FVIII: patients with normal FVIII (0.50–1.50 IU/mL) and patients with elevated FVIII (>1.50 IU/mL). This study was approved by the IRB at our center with waiver of informed consent.

FVIII levels were measured using spun plasma on the Siemens BCS XP instrument, an optical chromogenic method of detection. The studies are performed at the on-site coagulation laboratory. The laboratory reference range is 0.50–1.50 IU/mL. At our institution, FVIII levels >1.50 IU/mL are considered elevated; levels >2.00 IU/mL are considered severely elevated by our laboratory. In this study, all other thresholds used for laboratory values were based on the laboratory reference ranges.

### 2.2. Outcome Variables

Our primary outcome variable was any recurrent thromboembolic event, defined as the development of a new ischemic stroke, progressive stroke, MI, or DVT/PE during stroke service admission. Patients who experienced such events were retrospectively identified from our patient registry using definitions established prior to data abstraction [9, 11]. A new ischemic stroke was defined as a new vessel occlusion occurring outside of the distribution of the initial brain ischemic event detected on computed tomographic (CT) or magnetic resonance imaging (MRI) ordered in the setting of new neurological deficits during admission [12]. Progressive ischemic stroke was classified as the extension of the initial ischemic event identified on CT or MRI less than 24 hours following the development of neurological deterioration [12]. MI during admission was defined as troponin >1.0 *μ*g/L accompanied by electrocardiographic findings or clinical symptoms suggestive of cardiovascular thromboembolism [13]. We defined DVT/PE as an image-documented thrombus in the setting of clinical symptoms of venous thromboembolism (edema, acute respiratory insufficiency, and/or leg pain) during admission [14]. Secondary outcome variables included favorable discharge disposition (defined as discharge to home or acute inpatient rehabilitation compared to all others), poor functional outcome (defined as mRS 4–6) at discharge, and in-hospital mortality.

### 2.3. Statistical Analysis

Demographic and clinical data recorded throughout admission were compared between groups (FVIII 0.50–1.50 IU/mL and FVIII >1.50 IU/mL) using Chi square tests for categorical variables and *t*-tests or Wilcoxon Rank Sum tests when appropriate for continuous variables. Normality was assessed for all continuous variables. Crude and adjusted logistic regression analyses were conducted to assess the relationship between elevated FVIII and the outcomes of interest. Adjustment variables for the multivariable logistic regression models were chosen based on baseline differences between the two groups as well as clinical significance. As this was an exploratory analysis, no adjustments were made for multiple comparisons [15]. An alpha of 0.05 was set as the level of significance. All analyses were performed using SAS 9.3 (Cary, NC).

## 3. Results

Of 1,401 AIS patients, a total of 298 (19.9%) patients met inclusion criteria, with 203 (68.1%) having elevated FVIII levels (>1.50 IU/mL). 1,102 AIS patients were excluded for not having FVIII measured during stroke admission and 1 patient was excluded for age less than 18 at the time of hospitalization. The median time from being last seen normal to presentation was 5.9 hours (0.05 hours–654.9 hours). As shown in Table 1, a greater proportion of patients with elevated FVIII were black (79.3% versus 56.8%, *P* < 0.0001) and reported a history of diabetes (41.3% versus 17.0%, *P* < 0.0001). Patients with elevated FVIII had higher serum glucose on admission (6.72 versus 5.94 mmol/L, *P* = 0.0033) and had nearly double the frequency of cardioembolic AIS (19.3% versus 11.6%, *P* = 0.0007). Although there was no statistically significant difference in hospital mortality between patients with and without elevated FVIII (2.5% versus 0%, *P* = 0.1444), elevated FVIII patients were found to have a significantly higher frequency of new thrombotic events (18.7% versus 8.4%, *P* = 0.0218) when compared to patients with normal FVIII (Table 2). The frequency of new thrombotic events in this population of FVIII screened AIS patients did not differ significantly from the frequency of new thrombotic events in the population of patients in our registry whose FVIII levels were not screened during admission. The median time from admission to a recurrent thrombotic event was 5.1 days (range 0.01–26.9 days). When FVIII was analyzed as a continuous variable, we observed a significant positive correlation between FVIII and the occurrence of new thrombotic events (OR 1.01, 95% CI 1.00–1.01, *P* = 0.0002). This association remained even after adjusting for NIHSS on admission and TOAST classification (OR 1.01, 95% CI 1.00–1.01, *P* = 0.0013). We found for every 10-point increase in FVIII level that the odds of having the event, even after adjusting for covariates, increase by 10%. Additionally, median FVIII level during admission was found to be significantly higher in the patient group who experienced a recurrent thrombotic event compared to those who did not experience such an event (2.14 versus 1.71, *P* = 0.0003, Table 3).

Outcomes in patients with and without a new thrombotic event during stroke admission are displayed in Table 4. Although the frequency of in-hospital mortality was higher in patients with new thrombotic events compared to those without such events (4.4% versus 1.2%, *P* = 0.1252), this increased risk failed to reach statistical significance. Patients with new thrombotic events compared to those without such events had significantly higher frequencies of major disability (mRS at discharge of 4–6: 17.8% versus 3.2%, *P* < 0.0001). The crude logistic model shows an association between new thrombotic events and mRS at discharge of 4–6 (OR 6.54, 95% CI 2.31–18.5, *P* = 0.0004) and the association remains after adjusting for NIHSS on admission, FVIII, and TOAST criteria (mRS 4–6, OR 4.85, 95% CI 1.51–15.6, *P* = 0.0080).

## 4. Discussion

In our sample of ischemic stroke patients, we found that patients with elevated FVIII were more likely to experience recurrent thrombotic events during hospitalization, as compared to patients with normal FVIII levels. This association remained after adjusting for baseline NIHSS and TOAST classification. Moreover, patients with recurrent in-hospital thrombotic events had significantly higher rates of poor functional outcome compared to patients without secondary events. To our knowledge, this is the first study to evaluate FVIII levels and the frequency of recurrent thromboembolism in a sample of AIS patients.

Although the results of this present study suggest that elevated FVIII is associated with recurrent thrombotic events and, thus, worse functional outcome, the mechanism by which this occurs remains unclear. Previous work in AIS patients demonstrated that heightened elevated FVIII levels were associated with higher median erythrocyte sedimentation rate [7], suggesting that FVIII may serve as an acute phase reactant. Our study cannot distinguish patients with constitutional elevation from those with elevation of FVIII as an acute phase reactant, but our results support that FVIII is associated with acute thrombosis. However, other researches involving patients with venous thrombosis have demonstrated FVIII levels to be elevated both outside of the acute event [16] and independently of known acute phase reactants such as C-reactive protein [17], suggesting that elevations in FVIII may occur independently of the acute phase reaction in AIS. Further study is needed to determine if FVIII levels are persistently elevated after the acute phase of stroke and, if so, whether this phenomenon represents a risk factor for future recurrent thrombotic events.

While this study is unique in terms of evaluating FVIII levels and the frequency of recurrent thromboembolism in AIS patients, it is not without limitations. It is limited by its retrospective design, sample from a single stroke center, and relatively small sample size. Because of the exploratory nature of this study, sample size calculations were not calculated prior to the start of the study. We were able to detect clinically and statistically significant differences, as well as determining the prevalence of RTEs in order to conduct sample size calculations for future studies. Furthermore, missing data is an issue with retrospective studies; however, the missing data in this sample was less than 10% indicating that multiple imputation is not necessary.

Additionally, since FVIII was measured at the discretion of the treating physician based on clinical suspicion of hypercoagulable state in a patient presenting with AIS, FVIII levels in this cohort were neither universally nor randomly measured. While this reflects actual practice patterns, selection bias has the potential to distort the association we observed between FVIII levels and recurrent thrombotic events. Another potential limitation of this study is that, unlike in other studies on FVIII and ischemic stroke [18, 19], we measured FVIII in the acute setting which could have been iatrogenically augmented after intravenous thrombolysis. In our retrospective study, measurement was considered after the emergency management. Given that the half-life of FVIII is 8–12 hours, FVIII was measured in most cases after FVIII should have recovered from a potential interaction. Since the group with normal FVIII more often received tPA, if FVIII was “falsely” lowered by tPA, this should have made it more difficult to detect a difference in recurrent thrombotic event rates. This remains a major limitation and should be explored in prospective trials of FVIII collection before and after administration of tPA. Given our findings and previous work demonstrating similar relationships between high FVIII and recurrent thromboembolism in other populations, further study using large, diverse samples of AIS patients is needed to investigate whether or not FVIII may have the potential to serve as a clinically useful biomarker for the identification of patients at increased risk for new in-hospital thrombotic events following AIS.

## Figures and Tables

**Table 1:** Baseline characteristics of patients with and without elevated FVIII.

	Normal Factor VIII (0.50–1.50 IU/mL) (*N* = 95)	Elevated Factor VIII (>1.50 IU/mL) (*N* = 203)	*P* value
Age, median (range)	52 (26–85)	54 (19–90)	0.2908
Gender, % female	43 (45.3%)	108 (53.2%)	0.2015
Black race, %	54 (56.8%)	161 (79.3%)	<0.0001
Past medical history, %			
Coronary artery disease	18 (18.9%)	46 (22.7%)	0.4670
Diabetes	16 (17%)	83 (41.3%)	<0.0001
Hypertension	68 (72.3%)	152 (75.3%)	0.5940
Dyslipidemia	25 (26.6%)	76 (37.6%)	0.0625
Atrial fibrillation	2 (2.1%)	7 (3.5%)	0.5284
Prior stroke event	32 (33.7%)	88 (43.3%)	0.2129
Cancer	8 (8.4%)	11 (5.5%)	0.3284
Baseline NIHSS, median (range)	5 (0–24)	5 (0–33)	0.1666
Baseline laboratory values			
Glucose, median mmol/L (range)	5.94 (4.16–18.20)	6.72 (3.22–46.12)	0.0033
INR, median (range)	1 (0.7–3.8)	1 (0.8–3.1)	0.3625
IV tPA, %	47 (49.5%)	59 (29.1%)	0.0006
Active smoker, %	43 (46.2%)	69 (34.5%)	0.0543
Home medications, %			
Any antithrombotic	33 (34.7%)	74 (36.5%)	0.7735
Any antiplatelet	32 (33.7%)	69 (34.7%)	0.8673
Any anticoagulant	1 (1.1%)	8 (4.2%)	0.1275
TOAST			0.0007
Cardioembolic	11 (11.6%)	39 (19.3%)	
Large vessel disease	14 (14.7%)	34 (16.8%)	
Small vessel disease	14 (14.7%)	21 (10.4%)	
Cryptogenic (>1 cause)	3 (3.2%)	16 (7.9%)	
Cryptogenic (no cause found)	40 (42.1%)	44 (21.8%)	
Other	9 (9.9%)	45 (22.6%)	
Hospital medications, %			
Any inpatient antiplatelet	85 (89.5%)	189 (93.1%)	0.2833
Chemical DVT ppx	75 (78.9%)	159 (78.7%)	0.9632
Any full-dose anticoagulant	15 (15.9%)	57 (28.2%)	0.0221

NIHSS indicates National Institutes of Health Stroke Scale; IQR, interquartile range; INR, international normalized ratio; IV tPA, intravenous tissue plasminogen activator; TOAST, trial of ORG 10172 in Acute Stroke Treatment; DVT ppx, deep vein thrombosis prophylaxis. Prior stroke event was confirmed by either patient report or neuroimaging.

**Table 2:** Occurrence of new thrombotic events in patients with and without elevated FVIII.

	Normal Factor VIII (0.50–1.50 IU/mL) *N* = 95	Elevated Factor VIII (>1.50 IU/mL) *N* = 203	*P* value
Any new thrombotic event, %	8 (8.4%)	38 (18.7%)	0.0218
MI, %	1 (1.1%)	4 (2.0%)	0.3395
DVT/PE, %	1 (1.1%)	9 (4.6%)	0.0949
New AIS, %	1 (1.1%)	5 (2.5%)	0.2809
Progressive stroke, %	8 (8.4%)	22 (10.8%)	0.5183
In-hospital death, %	0	5 (2.5%)	0.1444

MI indicates myocardial infarction; DVT/PE, deep vein thrombosis or pulmonary embolism; AIS, acute ischemic stroke. Some patients experienced more than one thrombotic event; therefore, the number of patients with any new thrombotic event is not equal to the sum of individual thrombotic events.

**Table 3:** Baseline characteristics of patients with and without an in-hospital thrombotic event.

	Any new thrombotic event (*N* = 46)	No new thrombotic event (*N* = 252)	*P* value
FVIII level, median IU/mL (range)	2.14 (0.67–6.08)	1.71 (0.72–5.14)	0.0003
Age, median (range)	54 (19–90)	54 (21–85)	0.5778
Gender, % female	26 (56.5%)	125 (49.6%)	0.3881
Black race, %	35 (76.1%)	180 (71.4%)	0.5169
Past medical history, %			
Coronary artery disease	8 (17.4%)	56 (22.2%)	0.4631
Diabetes	15 (33.3%)	84 (33.6%)	0.9722
Hypertension	33 (75%)	187 (74.2%)	0.9115
Dyslipidemia	14 (31.8%)	87 (34.5%)	0.7269
Atrial fibrillation	2 (4.5%)	7 (2.8%)	0.5320
Stroke	17 (37%)	103 (40.9%)	0.0594
Cancer	6 (13.3%)	13 (5.2%)	0.0390
Baseline NIHSS, median (range)	5 (0–24)	5 (0–33)	0.1611
Baseline laboratory values			
Glucose, median mmol/L (range)	6.94 (3.89–22.53)	6.37 (3.22–46.12)	0.3710
INR	1.0 (0.9–2.5)	1.0 (0.7–3.8)	0.1310
IV tPA, %	12 (26.1%)	94 (37.3%)	0.1440
Active smoker, %	14 (31.8)	98 (39.4)	0.3428
Home medications, %			
Any antithrombotic	14 (30.4%)	93 (36.9%)	0.4003
Any antiplatelet	13 (28.9%)	88 (35.3%)	0.4016
Any anticoagulant	1 (2.4%)	8 (3.4%)	0.7398
TOAST, %			0.0014
Cardioembolic	6 (13%)	44 (18%)	
Large vessel disease	12 (26.1%)	36 (14.8%)	
Small vessel disease	1 (2.2%)	34 (13.9%)	
Cryptogenic (>1 cause)	9 (19.6%)	75 (30.7%)	
Cryptogenic (no cause found)	8 (17.4%)	11 (4.5%)	
Other	10 (21.7%)	44 (18%)	

NIHSS indicates National Institutes of Health Stroke Scale; IQR, interquartile range; INR, international normalized ratio; IV tPA, intravenous tissue plasminogen activator; TOAST, trial of ORG 10172 in Acute Stroke Treatment; DVT ppx, deep vein thrombosis prophylaxis.

**Table 4:** Outcomes in patients with and without an in-hospital thrombotic event.

	Any new thrombotic event (*N* = 46)	No new thrombotic event (*N* = 252)	*P* value
Favourable discharge disposition, %	11 (23.9%)	184 (73%)	<0.0001
In-hospital death, %	2 (4.4%)	3 (1.2%)	0.1252
Discharge mRS 4–6, %	8 (17.8%)	8 (3.2%)	<0.0001
Discharge mRS 5-6, %	3 (6.7%)	4 (1.6%)	0.0398

Favourable discharge disposition was defined as discharge to home or inpatient rehabilitation; mRS indicates modified Rankin Scale.

## References

[1] Kraaijenhagen R. A., In 'T Anker P. S., Koopman M. M. W., Reitsma P. H., Prins M. H., Van Den Ende A., Büller H. R. (2000). High plasma concentration of factor VIIIc is a major risk factor for venous thromboembolism. *Thrombosis and Haemostasis*.

[2] Koster T., Blann A. D., Briet E., Vandenbroucke J. P., Rosendaal F. R. (1995). Role of clotting factor VIII in effect of von Willebrand factor on occurrence of deep-vein thrombosis. *The Lancet*.

[3] Öner Erkekol F., Ulu A., Numanoglu N., Akar N. (2006). High plasma levels of factor VIII: an important risk factor for isolated pulmonary embolism. *Respirology*.

[4] Tanis B., Algra A., Van Der Graaf Y., Helmerhorst F., Rosendaal F. (2006). Procoagulant factors and the risk of myocardial infarction in young women. *European Journal of Haematology*.

[5] Meade T. W., Cooper J. A., Stirling Y., Howarth D. J., Ruddock V., Miller G. J. (1994). Factor VIII, ABO blood group and the incidence of ischaemic heart disease. *British Journal of Haematology*.

[6] Folsom A. R., Rosamond W. D., Shahar E. (1999). Prospective study of markers of hemostatic function with risk of ischemic stroke. The Atherosclerosis Risk in Communities (ARIC) Study Investigators. *Circulation*.

[7] Chang T. R., Albright K. C., Boehme A. K., Dorsey A., Sartor E. A., Kruse-Jarres R., Leissinger C., Martin-Schild S. (2014). Factor VIII in the setting of acute ischemic stroke among patients with suspected hypercoagulable state. *Clinical and Applied Thrombosis/Hemostasis*.

[8] Lopez A. D., Mathers C. D., Ezzati M., Jamison D. T., Murray C. J. (2006). Global and regional burden of disease and risk factors, 2001: systematic analysis of population health data. *The Lancet*.

[9] Siegler J. E., Boehme A. K., Dorsey A. M. (2013). A comprehensive stroke center registry: advantages, limitations, and lessons learned. *Medical Student Research Journal*.

[10] Adams H. P., Bendixen B. H., Kappelle L. J., Biller J., Love B. B., Gordon D. L., Marsh E. E. (1993). Classification of subtype of acute ischemic stroke: definitions for use in a multicenter clinical trial. *Stroke*.

[11] Albright K. C., Martin-Schild S., Bockholt H. J. (2011). No consensus on definition criteria for stroke registry common data elements. *Cerebrovascular Diseases Extra*.

[12] Siegler J. E., Boehme A. K., Albright K. C., George A. J., Monlezun D. J., Beasley T. M., Martin-Schild S. (2013). A proposal for the classification of etiologies of neurologic deterioration after acute ischemic stroke. *Journal of Stroke and Cerebrovascular Diseases*.

[13] McNamara R. L., Brass L. M., Drozda J. P., Go A. S., Halperin J. L., Kerr C. R., Lévy S., Malenka D. J., Mittal S., Pelosi F., Rosenberg Y., Stryer D., Wyse D. G., Radford M. J., Goff D. C., Grover F. L., Heidenreich P. A., Peterson E. D., Redberg R. F. (2004). ACC/AHA key data elements and definitions for measuring the clinical management and outcomes of patients with atrial fibrillation: a report of the American College of Cardiology/American Heart Association Task Force on clinical data standards (writing commitee to develop data standards on atrial fibrillation). *Journal of the American College of Cardiology*.

[14] Ramzi D. W., Leeper K. V. (2004). DVT and pulmonary embolism: part I. Diagnosis. *American Family Physician*.

[15] Bender R., Lange S. (2001). Adjusting for multiple testing—when and how?. *Journal of Clinical Epidemiology*.

[16] O'Donnell J., Mumford A. D., Manning R. A., Laffan M. (2000). Elevation of FVIII:C in venous thromboembolism is persistent and independent of the acute phase response. *Thrombosis and Haemostasis*.

[17] Kamphuisen P. W., Eikenboom J. C. J., Vos H. L., Pablo R., Sturk A., Bertina R. M., Rosendaal F. R. (1999). Increased levels of factor VIII and fibrinogen in patients with venous thrombosis are not caused by acute phase reactions. *Thrombosis and Haemostasis*.

[18] Gustafsson C., Blomback M., Britton M., Hamsten A., Svensson J. (1990). Coagulation factors and the increased risk of stroke in nonvalvular atrial fibrillation. *Stroke*.

[19] Landi G., D'Angelo A., Boccardi E., Candelise L., Mannucci P. M., Orazio E. N., Morabito A. (1987). Hypercoagulability in acute stroke: prognostic significance. *Neurology*.

